# ASO Author Reflections: Survival Trends in Gastric Adenocarcinoma

**DOI:** 10.1245/s10434-018-6844-4

**Published:** 2018-10-08

**Authors:** Johannes Asplund, Jesper Lagergren

**Affiliations:** Upper Gastrointestinal Surgery, Department of Molecular Medicine and Surgery, Karolinska Institutet and Karolinska University Hospital, Stockholm, Sweden

## Past

Gastric cancer (mainly adenocarcinoma) is common and has a poor prognosis. By causing approximately 834,000 deaths annually, it is presently the third most common cancer-related death globally.^1^ Surgery is the mainstay in the curative treatment of gastric adenocarcinoma, and for locally advanced disease, perioperative chemotherapy is usually added to surgery.^2^ Gastric adenocarcinoma often is subclassified anatomically into noncardia (“distal”) or cardia (“proximal”) location because of the major differences in risk factor profiles and incidence trends of these locations.^2^ The prognosis in noncardia adenocarcinoma is generally better than that in cardia adenocarcinoma, but a declining survival for noncardia adenocarcinoma and an increasing survival for cardia adenocarcinoma has recently been observed.^3^^,^^4^ This study was designed to assess changes in survival in gastric adenocarcinoma over time by assessing overall survival, survival in patients selected for surgery, and in those who do not undergo surgery.^5^ This is important for evaluating potential changes in the detection and treatment of this tumor.

## Present

The present study included all patients with gastric noncardia or cardia adenocarcinoma recorded in the 98% complete Swedish Cancer Registry in 1990–2013, and they were followed up until 2017.^5^ Despite a decreased proportion of patients who underwent resectional surgery, the overall survival remained unchanged for gastric noncardia adenocarcinoma and improved for cardia adenocarcinoma.^5^ During the last period, the 5-year relative survival was 18% for both subsites of gastric adenocarcinoma in Sweden. The postoperative 5-year relative survival increased for both noncardia and cardia adenocarcinoma, which is likely explained by stricter selection of patients for surgery. In patients who did not undergo surgery, the 5-year relative survival was very low (2–5%).

## Future

In Sweden, the surgical treatment of cardia adenocarcinoma has been increasingly centralized to larger centers, but this development has been absent or slower for gastric noncardia adenocarcinoma. The lack of improvement in the survival in noncardia adenocarcinoma despite a clearly improved survival in cardia adenocarcinoma might indicate a benefit of centralization also of the treatment of noncardia adenocarcinoma. Trends in the overall and postoperative long-term prognosis in gastric cancer need to be assessed continuously, ideally using registries with complete case detection and follow-up, i.e., similar to the present study. Such studies can help to monitor the results of health care strategies, e.g., centralization of services, and help to identify needs for new strategies to improve the treatment and survival of this deadly cancer.
